# Active Secondary Metabolites from Root-Associated Endophytic Fungus *Aspergillus tubingensis* ZMGR14 and Their Activities Against Plant Pathogenic Fungi

**DOI:** 10.3390/biology15100812

**Published:** 2026-05-21

**Authors:** Haoyue Liu, Hui Jin, Xiaoyan Yang, Zhongxiang Xu, Jinchun Cheng, Lihong Wang, Zuhua Yan, Bo Qin

**Affiliations:** 1 College of Advanced Agriculture and Life Sciences, Weifang University, Weifang 261061, China; 20230045@wfu.edu.cn; 2 Research Center for Natural Medicine and Chemical Metrology, Lanzhou Institute of Chemical Physics, Chinese Academy of Sciences (CAS), Lanzhou 730000, China; yangxiaoy@licp.cas.cn (X.Y.); yanzuhua21@mails.ucas.ac.cn (Z.Y.); 3 Animal, Plant & Food Inspection Center of Nanjing Customs, Nanjing 210019, China; xuzhongxiang@customs.gov.cn; 4 Chemical Engineering College, Northwest Minzu University, Lanzhou 730030, China; chengjinchun@foxmail.com; 5 Third Reserve Assets Administration, Lanzhou Administration Station, Lanzhou 730020, China; liuhaoyue0820@163.com

**Keywords:** *Aspergillus tubingensis* ZMGR14, secondary metabolites, antifungal activity, plant pathogenic fungi, EtOAc extract

## Abstract

This study aimed to separate and characterize compounds from *Aspergillus tubingensis* ZMGR14. The antifungal activities of monomer compounds and the ethyl acetate (EtOAc) layer from the fermented liquor of *A. tubingensis* were isolated, purified and structurally identified. The EtOAc layer from the fermented liquor showed significant antifungal activity against *Fusarium oxysporum* and *Alternaria alternata* with IC_50_ values of 273.8 and 330.7 μg·mL^−1^, respectively. The EtOAc extract was further purified by column chromatography and recrystallization to yield six compounds. Antifungal trials showed that Cyclo-(L-Pro-D-Leu) (**5**) exhibited the highest inhibition against *A. alternata* and *F. oxysporum*, with an IC_50_ value of 48.1 and 232.7 μM, respectively, and cyclo-(L-Pro-L-Leu) (**6**) displayed moderate antifungal activity against *Alternaria solani*, with an IC_50_ value of 493.4 μM. The results suggest that the EtOAc extract of ZMGR14 and its bioactive compounds hold promise as environmentally friendly microbial fungicides.

## Simple Summary

An endophytic fungus labeled as ZMGR14 was isolated from the root of *Stipa purpurea* and identified as *Aspergillus tubingensis*. This study aims to isolate and characterize compounds from the secondary metabolites of *A. tubingensis* ZMGR14 and to investigate their antifungal activities. The results showed that the ethyl acetate (EtOAc) layer from the fermented liquor showed significant antifungal activity against *Fusarium oxysporum* and *Alternaria alternata*. The EtOAc extract was further purified by column chromatography and recrystallization to yield six compounds. For Compounds **2**, **5**, and **6**, which belong to the diketopiperazines (DKPs), different activities were detected as the position of substituents changed. Antifungal trials showed that Cyclo-(L-Pro-D-Leu) (**5**) exhibited the highest inhibition against *A. alternata* and *F. oxysporum*, with an IC_50_ value of 48.1 and 232.7 μM, respectively, and cyclo-(L-Pro-L-Leu) (**6**) displayed moderate antifungal activity against *Alternaria solani*, with an IC_50_ value of 493.4 μM. The relative configuration of 3-H and the numbers of hydroxyl substitutions in the structures of the DKPs are likely critical factors responsible for the antifungal activity against *A. alternata, A. solani* and *F. oxysporum*. The present results indicated that the EtOAc extract of ZMGR14 and its bioactive compounds can be used as a promising microbial fungicide.

## 1. Introduction

Plant pathogenic fungi are among the major pathogen microorganisms that cause plant diseases [1]. Currently, control of microbial disease has depended chiefly on synthetic fungicides, such as carbendazim, scala, mancozeb and so on. However, synthetic fungicides usually produce chemical residues due to their high toxicity and difficulty in degrading, which become potential environmental pollutants [2]. As a result, their use has been limited and gradually eliminated [3]. We urgently need to develop effective, safe, and low-resistant alternatives from plants, microorganism and other natural sources. Exploitation of fungicides from microorganisms is considered to be a feasible options, which meets the requirements of biological control. Fungal endophytes or their products exhibit a number of chemical and biological advantages as fungicides [4,5]. Efficient biodegradability is an important advantage of microbial metabolites, which always degrade within a month or even a few days, so they cause low residue and little harm to the natural environment [6]. Another important advantage comes from their strict selectivity and specificity [7,8]. Ideally, fungicides only have prevention and cure effects on the infection of pathogenic fungi without toxic effects on host plants and other organisms.

Plant-derived *Aspergillus* is a large class of plant micro-organisms. Among the endophytic fungi, the genus *Aspergillus* in Ascomycota deserves special attention for its biological activity [9,10]. Previous chemical studies of *Aspergillus* have reported the separation of some bioactive natural products, such as alkaloids, cyclopeptide, steroids polyketides and terpenes [11,12,13]. Its potential as a biological fungicide has been demonstrated in combating the presence of multiple fungal pathogens, including several species of *Fusarium solanum*, *Fusarium oxysporum* [14] and *Botrytis cinerea*, which are particularly common in plant tissue and soil, and seriously endanger the yield of crops [15]. Some of these compounds show significant antibacterial activities, while others show anticancer or antioxidant activities [16]. In addition, it has also been found that the compounds produced by endophytic fungi are sometimes the same as those produced by their respective hosts, which have been only isolated from higher plants [17]. One of the main chemical components of *Forsythia suspensa* is lignan such as phillyrin and forsythiaside [8]; it is demonstrated that phillyrin is also detected in the endophytic fungus *Colletotrichum gloeosporioides*, which is isolated from *F. suspensa* [18].

*Stipa purpurea* is a dominant forage grass widely distributed in the alpine grassland in the Qinghai–Tibet Plateau. Because of its high crude protein content, less crude fiber, and relatively rich nutritional value, it is highly preferred by livestock [19]. *S. purpurea* possesses strong capabilities of cold and drought tolerance and disease resistance. In the present study, an endophytic fungus labeled as ZMGR14 was isolated from the root of *S. purpurea* and identified as *A. tubingensis* based on the morphological characteristics and ITS rDNA sequence. The objective of this study was to separate and characterize compounds associated with ZMGR14 that could potentially be used for their antifungal activity against pathogenic fungi. To the best of our knowledge, this is the first report on the composition and antifungal activity of secondary metabolites of the *A. tubingensis* isolated from *S. purpurea*.

## 2. Materials and Methods

### 2.1. General Experimental Procedures

The NMR spectral data were collected on a Varian Mercury-400BB instrument (Varian Inc., Palo Alto, CA, USA) (^1^H NMR; 400 MHz, ^13^C NMR; 101 MHz) and Ascend 600/Avance NEO instrument (Bruker BioSpin, Rheinstetten, Germany) (^1^H NMR; 600 MHz, ^13^C NMR; 151 MHz) with TMS as the internal standard. The HR-MS data were obtained using a Bruker micrOTOF-QII instrument (Bruker Daltonics, Billerica, MA, USA). Column chromatography (CC) and thin-layer chromatography (TLC) were performed on silica gel and precoated silica gel GF254 plates, respectively (Qingdao Marine Chemical Factory, Qingdao, China). Sephadex LH-20 (Amersham Pharmacia, Uppsala, Sweden) was used for CC.

### 2.2. Plant Materials and Endophytic Fungus

The fungal strain *A. tubingensis* ZMGR14 was isolated from the roots of *S. purpurea*, which was collected from Gansu Province in June 2016. According to the methods in our previous report [20], the strain was identified by its morphological characteristics and molecular (ITS and 18S rDNA sequence) analyses. The sequence of the strain (GenBank Accession No. MT446066) showed up to 100.00% similarity to the known *A. tubingensis* isolate AJH1 (GenBank Accession No.MT498681.1), and phylogenetic analyses were estimated in MEGA 6.0 (Figure S1).

### 2.3. Fermentation, Extraction and Isolation

The *A. tubingensis* ZMGR14 was fermented in potato dextrose broth at 28 °C on a rotary (120 rpm) shaker for 7 days. The fermentation broth (100 L) was filtered to harvest the cultures, which were extracted with EtOAc (100 L × 3 times). The EtOAc extract (about 25 g) was chromatographed on a D-101 macroporous resin column using gradient elution of methanol and H_2_O (MeOH/H_2_O 25:75 to 100:0) to yield four fractions (Fr.A-D) (Table S1). Fr.A was subjected to silica gel CC, eluted with a gradient of petroleum ether and acetone (PE/acetone, 8:1–1:1) to afford Fr.A-10 and Fr.A-12, respectively. Fr.A-10 was separated on silica-gel CC and eluted with dichloromethane-ethyl acetate (CH_2_Cl_2_/EtOAc, 7:1, *v*/*v*) to give Compound **1** (170.5 mg). Fr.A-12 was purified by a Sephadex LH-20 column chromatography eluted with MeOH to afford Compound **2** (27.6 mg). Fr.B was separated by CC of silica-gel (PE/EtOAc, 20:1–0:1) to give 14 subfractions. Fr.B-6 and Fr.B-12 were subjected to a Sephadex LH-20 column, and these were eluted with methanol to obtain Compound **3** (202.3 mg) and **4** (5.5 mg), respectively. The Fr.B-14 fraction was subjected to CC of silica gel (CH_2_Cl_2_/MeOH, 100:1 to 5:1) to afford Fr.B-14-1 and Compound **5** (113.2 mg). Fr.B-14-1 was passed through a Sephadex LH-20 column with MeOH as eluent to afford Compound **6** (34.4 mg).

### 2.4. Antifungal Bioassays

The antifungal activities of EtOAc extract, purified compounds, and two positive controls, allicin [21] and kasugamycin [22], against three common plant pathogens (*Fusarium oxysporum*, *Alternaria solani* and *Alternaria alternata)* were studied. The EtOAc extract was detected at the concentrations of 25, 50, 100, 200, 400 and 800 μg·mL^−1^, and the five purified compounds (since the amount of isolated Compound **4** was little, it was hard to carry out the antifungal activity experiment) were detected at the concentrations of 25, 50, 100, 200 and 400 μM. Different concentrations of the samples were prepared with dimethyl sulfoxide (DMSO) and diluted with PDA medium. DMSO content in each dish never exceeded 1% of its volume. Inoculate the isolated pathogenic fungi in the center of a PDA plate containing samples, incubate at 28 °C for 7 days, repeat three times, and measure the diameters of the pathogenic fungi. Equal amounts of DMSO were used as the untreated controls. Percent inhibition was calculated by the formula *I* (%) =$\;\frac{C-T}{C}$ × 100 where C = radial growth of fungus in control; T = radial growth of fungus in the treatment.

### 2.5. Statistical Analysis

All statistical analysis were completed by using SigmaPlot 12.0 (Systat Software, 218, San Jose, CA, USA). All data are presented as mean ± standard deviation (SD). Statistical differences in mycelial radial growth between the treatment groups and the DMSO-treated control group were evaluated using one-way analysis of variance (ANOVA) followed by Tukey’s honestly significant difference (HSD) post hoc test for pairwise comparisons. A *p*-value of less than 0.05 was considered statistically significant for all analyses. The half-maximal inhibitory concentration (IC_50_) values were calculated by SPSS (v16.0).

## 3. Results

### 3.1. Antifungal Activity of EtOAc Extract of A. tubingensis

**Figure 1:**
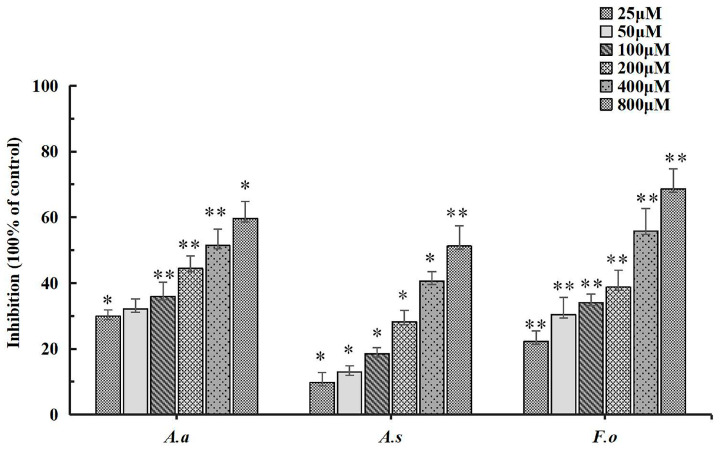
Antifungal activities of EtOAc extract against *A. alternata* (*A.a*), *A. solani* (*A.s*) and *F. oxysporum* (*F.o*). The y-axis represents the percentage inhibition of mycelial radial growth compared to the untreated control. Each value represents the mean ± standard deviation of two runs with three replicates. * indicates a significant difference at *p* < 0.05; ** indicates a remarkable difference at *p* < 0.01.

In the bioassay-guided isolation of antifungal compounds from *A. tubingensis*, it is necessary to test the EtOAc extract for antifungal activity. Figure 1 shows the antifungal activities of the EtOAc extract of *A. tubingensis* against *A. alternata*, *A. solani* and *F. oxysporum*. The EtOAc extract showed the highest inhibition rate against *F. oxysporum* at 800 μM, which was 68.64%, followed by *A. alternata* (59.63%), and *A. solani* (51.25%). The inhibition rate showed the same increasing trend as concentration, which recorded a significant difference (*p* < 0.05). At the same time, the extract exhibited slightly dose-dependent antifungal activity toward *A. alternata*, *A. solani* and *F. oxysporum*. The EtOAc extract was the most active against *F. oxysporum*, with an IC_50_ value of 273.8 μg·mL^−1^, followed by *A. alternata* and *A. solani* with an IC_50_ value of 330.7 μg·mL^−1^ and 743.1 μg·mL^−1^, respectively (Table 1). The antifungal activity of *A. tubingensis* EtOAc extract against *A. alternata*, *A. solani* and *F. oxysporum* is reported for the first time in the present study.

### 3.2. Isolation and Identification of Compounds from A. tubingensis

**Figure 2:**
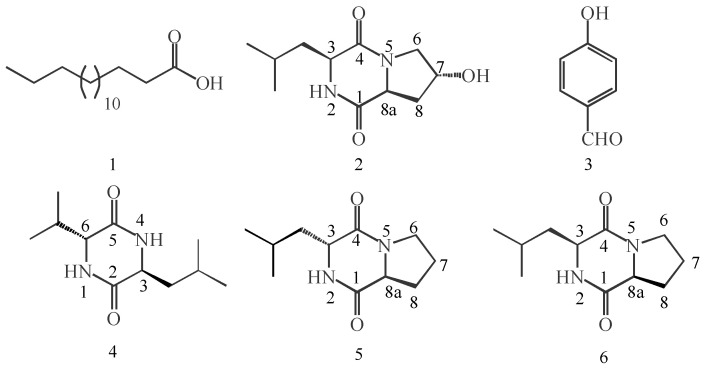
Chemical structures of Compounds **1** to **6**. The compounds were identified as hexadecanoic acid (**1**), cyclo-[L-leucine-L-(4R-hydroxyprolinyl)] (**2**), *p*-hydroxy benzaldehyde (**3**), cyclo-(L-Leu-D-Val) (**4**), cyclo-(L-Pro-D-Leu) (**5**), cyclo-(L-Pro-L-Leu) (**6**).

Six active compounds were isolated from the EtOAc extract of *A. tubingensis*, all of which were known to science previously (Figure 2). The spectroscopic data are summarized in Supplementary Text S1.

### 3.3. Antifungal Activity of the Compounds from A. tubingensis

**Figure 3:**
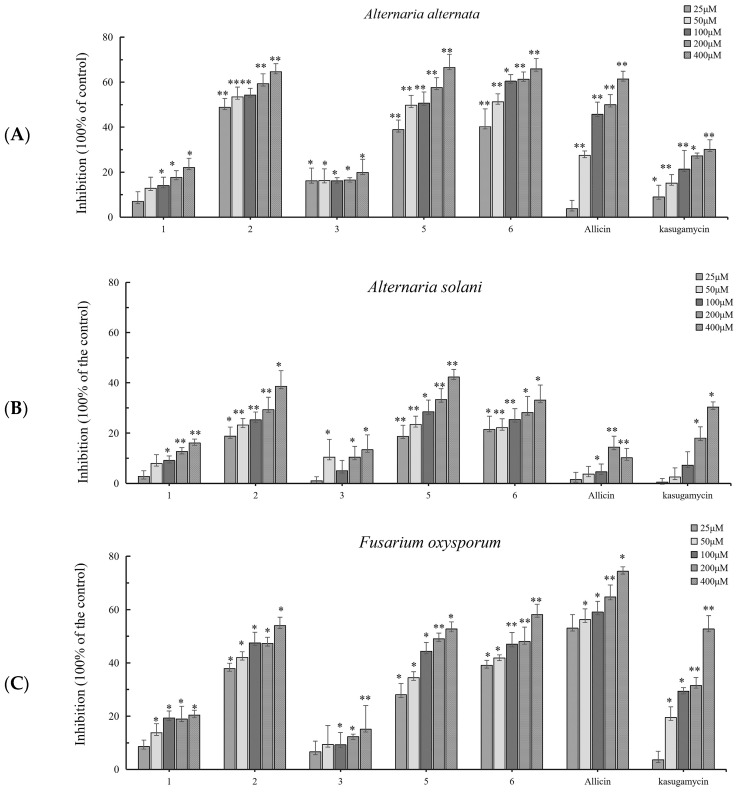
Antifungal activities of five compounds, allicin and kasugamycin against *A. alternata* (**A**), *A. solani* (**B**) and *F. oxysporum* (**C**). Each value represents the mean ± standard deviation of two runs with three replicates. * indicates a significant difference at *p* < 0.05; ** indicates a remarkable difference at *p* < 0.01.

**Figure 4:**
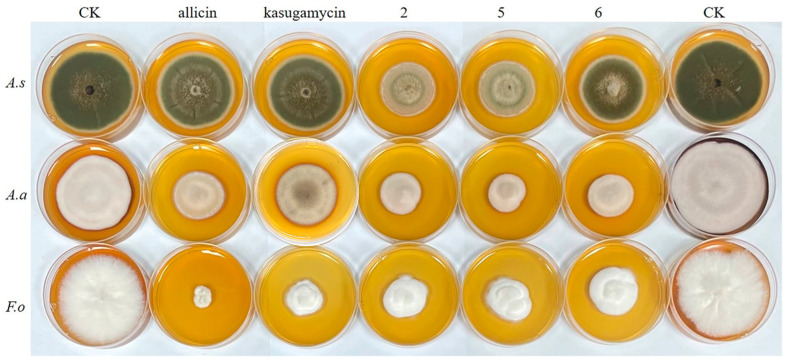
Antifungal activities of Compounds **2**, **5**, **6** and allicin and kasugamycin against *A. alternata* (*A.a*), *A. solani* (*A.s*) and *F. oxysporum* (*F.o*) at the concentration of 400 μM. CK, the control group. Each experiment was performed with three replicates.

The antifungal activity of five compounds and positive controls was initially tested at a concentration range of 50 μM to 400 μM and the results are given in Figure 3 and Figure 4. Three of these five compounds, cyclo-[L-leucine-L-(4R-hydroxyprolinyl)] (**2**), cyclo-(L-Pro-D-Leu) (**5**) and cyclo-(L-Pro-L-Leu) (**6**), showed obvious antifungal activity, with inhibition values of ≥50% against *F. oxysporum* at a concentration of 100 μM (Figure 3C). As shown in Figure 3A, Compounds **2**, **5** and **6** produced significant activity (60–70% inhibition rate) at a concentration of 800 μM against *A. alternata*. However, these three compounds were observed to exhibit selective moderate antifungal activity against *A. solani* (Figure 3B). Under the same conditions, the growth inhibition of the remaining two compounds were relatively low or no activity. Compared with commercial fungicides, the antifungal activity of Compounds **2**, **5**, and **6** against *A. alternata* and *A. solani* wa strongers than that of kasugamycin and allicin, but the antifungal activity toward *F. oxysporum* was slightly weaker than that of allicin. Compounds **1**–**6** and kasugamycin and allicin were tested at a series of concentrations to assess their IC_50_ values (Table 1). The antifungal activities of Compounds **2**, **5** and **6** against *A. alternata*, *A. solani* and *F. oxysporum* have similar variation patterns; Compound **6** has the lowest IC_50_ value of 48.1 μM against *A. alternata*. The inhibitory activities of Compounds **2** and **5** against three pathogenic fungi were higher than those of kasugamycin. Figure 4 shows the inhibitory activity of Compounds **2**, **5**, **6** and kasugamycin and allicin against three pathogenic fungi at the concentration of 400 μM. The results show that the skeleton of the 2,5-diketopiperazine Compounds **2**, **5** and **6** has the potential to inhibit the growth of pathogenic fungi.

## 4. Discussion

So far, many studies have indicated that diketopiperazines (DKPs) isolated from plant, fungi, actinomycetes and bacteria, and their biological functions, have attracted more and more attention, showing various bioactivities, including antibacterial [23], antifungal [24], antitumor [25], quorum quenching [26] and insecticidal [12] activities. DKPs are the most widely distributed cyclic dipeptides in nature and form a stable six-membered ring skeleton via cyclization of two amino acids through peptide bonds [27]. In a systematic review of the research progress on DKP compounds over the past decade, it is indicated that DKPs can also act as signaling molecules in intercellular communication involved in quorum sensing regulation, and are consequently regarded as important lead compounds with the potential for development into novel anti-infective agents [28,29]. In recent years, large-scale genome analysis has revealed the diversified evolutionary patterns in DKP biosynthesis in fungi [30].

Antifungal DKPs have also been isolated from various fungal species. For example, DKPs produced by *A. alternata* have been shown to inhibit *Plasmopara viticola* on grapevine leaves [31], and genome mining revealed that DKPs are essential for the antifungal activity of *Epicoccum dendrobii* against *B. cinerea* [32]. These findings have laid an important foundation for further exploration of specific types of natural products [33]. For the compounds of DKPs (**2**, **5**, **6**) with the position of the substituent changing, different activity was detected. The structural diversity and broad-spectrum bioactivities of DKPs have been systematically summarized in a recent comprehensive review [27]. In this work, four DKP compounds were obtained from ZMGR14. The test of Compound 4 against pathogenic fungus failed due to the limited amount. All compounds (**2**, **5**, **6**) displayed significant antifungal activity against *A. alternata*, *A. solani* and *F. oxysporum*, especially cyclo-(L-Pro-L-Leu) (**6**) against *F. oxysporum* (IC_50_ = 48.1 μM).

It was found that both cyclo-[L-leucine-L-(4R-hydroxyprolinyl)] (**2**) and cyclo- (L-Pro-L-Leu) (**6**), with only one hydroxyl substitution difference, showed different antifungal activities against *A. alternata* and *A. solani* in this study (Table 1). Compound **6** exhibited stronger activity against *A. alternata* (IC_50_ = 48.1 μM) than Compound **2** (IC_50_ = 125.7 μM), and this difference may be attributed to the hydroxyl substitution in Compound 2. In contrast, the antifungal activity of Compound **2** (IC_50_ = 272.5 μM) was equivalent to that of Compound **6** (LC_50_ = 232.7 μM) against *F. oxysporum*. The difference in antifungal activity between Compounds **5** and **6** might arise from their relative configurations at 3-H: cis in Compound **6** and trans in Compound **5** (Figure 2). In addition, the activity of Compound **6** was stronger than that of Compound **5** against *A. alternata* and *A. solani*. In a previous study on endophytic metabolites from a Rhabditidae entomopathogenic nematode, the relative configuration of 3-H was thought to be an important factor responsible for antibacterial and antifungal activity [34]. It was also found that Compound **6** (MIC = 4 μM) was stronger than Compound **5** (MIC = 8 μM) against the growth of the pathogen *Penicillium expansum*, with the result similar to our study. Studies have shown that Compound **6** directly inhibits fungal growth and also activates plant defense by inducing PR-1 and PDF1.2 in *Arabidopsis thaliana* [32]. Compared to the positive controls, Compound **6** showed superior activity against *A. alternata* (IC_50_ = 48.1 μg/mL) relative to allicin (257.3 μg/mL) and kasugamycin (641.6 μg/mL). Against *F. oxysporum*, Compound **6** (IC_50_ = 232.7 μg/mL) was comparable to kasugamycin (344.0 μg/mL) but less active than allicin (18.5 μg/mL). These results suggest that Compound **6** is a promising lead for further development as a botanical fungicide.

However, Compound **5** has good antibacterial activity against *Bacillus subtilis*, *Staphylococcus aureus*, *Escherichia coli* and *Pseudomonas aeruginosa*, while Compound **6** has good antibacterial activity against only Gram-positive bacteria [35]. Kim et al. found that among the DKPs produced by *Pseudomonas sesami* BC42, both cyclo(L-Leu-L-Pro) and cyclo(D-Leu-D-Pro) exhibited significant antifungal activity against *Colletotrichum orbiculare*, whereas cyclo(D-Leu-L-Pro) showed no activity [36]. Kumar et al. found that Compound **5** exhibited significantly inhibitory activity against important agricultural pathogenic fungi, including *F. oxysporum*, *R. solani*, and *P. expansum*, compared to the commercial fungicide Bavistin [37].

These results collectively indicate that chiral amino acid residues and stereochemical configurations play a key role in the biological activity of DKPs. The inhibitory effect of Compound **5** on the production of aflatoxin by *Aspergillus parasitic* has recently been reported [38]. Similarly, Begum et al. [39] reported that the antifungal metabolite of fluorescent *Pseudomonas fluorescens* was DKP, which showed significant antifungal activity at 1000 ppm (*p* < 0.01), and the compound can be used for the prevention and control of plant diseases, especially sorghum cereal mold, under field conditions. The invitro IC_50_ values of the active DKPs in this study ranged from 48.1 to 272.5 μM. A previous greenhouse study reported that DKPs at 10^−3^–10^−6^ M effectively inhibited *Plasmopara viticola* sporulation on grapevine leaves without causing phytotoxicity [32]. Accordingly, we propose that DKP concentrations of 50–200 μM are a suitable starting range for future in planta trials, with further optimization depending on target pathogens and crop systems.

## 5. Conclusions

The antifungal activities of isolated compounds and the ethyl acetate layer from the fermentation broth of *A. tubingensis* ZMGR14 were obtained in this study. The relative configuration of 3-H and the numbers of hydroxyl substitutions in the structures of the DKPs are likely critical factors responsible for the antifungal activity against *A. alternata*, *A. solani* and *F. oxysporum*. The present results indicated that the EtOAc extract of ZMGR14 and its bioactive compounds can be used as a promising microbial fungicide.

## Figures and Tables

**Table 1:** IC_50_ values of EtOAc extract, five compounds, allicin and kasugamycin against three pathogenic fungi.

Compound	IC_50_ (95% CL) (μg/mL)		
	*A. alternata*	*A. solani*	*F. oxysporum*
EtOAc extract	330.7 (213.9–703.7)	743.1 (520.2–1.3 × 10^3^)	273.8 (201.1–404.6)
**1**	872.2 (586.1–2.3 × 10^3^)	898.0 (617.0–2.1 × 10^3^)	1.2 × 10^3^ (-^b^)
**2**	146.1 (125.7–283.9)	587.9 (418.7–1.2 × 10^3^)	272.5 (152.1–1.2 × 10^3^)
**3**	2.5 × 10^3^ (-^b^)	1.0 × 10^3^ (-^b^)	1.2 × 10^3^ (708.4–3.9 × 10^4^)
**5**	112.1 (13.9–183.1)	493.4 (369.4–844.5)	298.1 (216.3–500.7)
**6**	48.1 (-^b^)	840.4 (503.8–9.0 × 10^3^)	232.7 (136.1–480.4)
Allicin	257.3 (-^b^)	948.4 (-^b^)	18.5 (17.7–52.1)
Kasugamycin	641.6 (-^b^)	495.7 (353.5–1.6 × 10^3^)	344.0 (-^b^)

“IC_50_” indicates the concentration required to inhibit fungal growth by 50%; “95% CL” indicates the 95% confidence limit; and “b” indicates that the confidence interval could not be calculated reliably.

## Data Availability

All data generated or analyzed during this study are included in this published article.

## Supplementary Materials

The following supporting information can be downloaded at https://www.mdpi.com/article/10.3390/biology15100812/s1, Figure S1: Phylogenetic trees based on neighbor-joining (NJ) analysis of the ITS sequences of the ZMGR14 strain; Table S1: Separation and purification of ethyl acetate extract using D-101 macroporous resin column and mobile phase system (MeOH/H_2_O). Text S1: Spectroscopic data of compounds **1**–**6**.

## Abbreviations

The following abbreviations are used in this manuscript:

ANOVA	One-way analysis of variance
C	Radial growth of fungus in control
CC	Column chromatography
CH_2_Cl_2_	Dichloromethane
CL	Confidence Limit
CK	The control group
DMSO	Dimethyl sulfoxide
DPPH	1,1-diphenyl-2-picryhydrazyl
EtOAc	Ethyl acetate
HSD	Honestly significant difference
IC_50_	Half-maximal inhibitory concentration
MeOH	Methanol
MIC	Minimum inhibitory concentration
PE	Petroleum ether
TLC	Thin-layer chromatography
SD	Standard deviation
