# Supplementary material for: Active Secondary Metabolites from Root-Associated Endophytic Fungus Aspergillus tubingensis ZMGR14 and Their Activities Against Plant Pathogenic Fungi
Source: Biology (Basel). 2026 May 21;15(10):812. doi: 10.3390/biology15100812 (PMC13203383; doi:10.3390/biology15100812)
Supplement: Supplementary file 1 [file biology-15-00812-s001.zip › Table S1.pdf]

Table S1 Separation and purification of ethyl acetate extract using D-101 macroporous resin column and mobile phase system (MeOH/H_2O)

Mobile phase composition (MeOH/H_2O, v/v)	Fraction	Weight (g)
25:75	Fr.A	4.0
50:50	Fr.B	10.0
75:25	Fr.C	5.0
100:0	Fr.D	3.0
