# Supplementary material for: Active Secondary Metabolites from Root-Associated Endophytic Fungus Aspergillus tubingensis ZMGR14 and Their Activities Against Plant Pathogenic Fungi
Source: Biology (Basel). 2026 May 21;15(10):812. doi: 10.3390/biology15100812 (PMC13203383; doi:10.3390/biology15100812)
Supplement: Supplementary file 1 [file biology-15-00812-s001.zip › Text S1.pdf]

**Text S1**: Spectroscopic data of compounds 1-6.

**Hexadecanoic acid (1)**: $C_{16}H_{32}O_2$ was obtained as white crystal, ESIMS: m/z 279.2295 $[M+Na]^+$. $^1H$ NMR (600 MHz, DMSO) δ 4.88 (s, 1H, OH-1), 2.27 (t, *J*=7.2 Hz, 2H, H-2), there are multiple H protons at 1.37-1.18 (m, 26H), 0.90 (t, *J*=6.9 Hz, 3H, H-16). $^{13}C$ NMR (151 MHz, DMSO) δ177.66(C-1), 34.97(C-2), 33.10(C-14), 30.82(C-11,12), 30.80(C-13), 30.75(C-7), 30.65(C-10), 30.51(C-4,5,6), 30.47(C-8), 30.28(C-9), 26.11(C-3), 23.76(C-15), 14.48(C-16).

**Cyclo[L-leucine-L-(4R-hydroxyprolinyl)] (2)**: $C_{11}H_{18}O_3N_2$ was obtained as colorless solid, $[\alpha]^{20}_D$ -64° (c 0.1, $CH_3OH$), ESIMS: m/z 249.1210 $[M+Na]^+$. $^1H$ NMR (400 MHz, $CD_3OD$) δ 4.51 (dd, *J*=10.1, 6.6 Hz, 1H, H-8a), 4.45 (m, 1H, H-7), 4.18 (m, 1H, H-3), 3.65 (dd, *J*=12.8, 4.4 Hz, 1H, H-6a), 3.42 (d, *J*=12.8 Hz, 1H, H-6b), 2.27 (m, 1H, H-8b), 2.08 (m, 1H, H-8a), 1.97-1.82 (m, 2H), 1.55-1.45 (m, 1H), 0.96 (d, *J*=1.9 Hz, 3H), 0.95 (d, *J*=1.7 Hz, 3H). $^{13}C$ NMR (101 MHz, $CD_3OD$) δ 173.06 (C-1), 169.04 (C-4), 69.11 (C-7), 58.70 (C-8), 55.16 (C-3), 54.59 (C-6), 39.38, 38.15 (C-8), 25.78, 23.30, 22.19 .

***p*-hydroxy benzaldehyde (3)**: $C_7H_6O_2$ was obtained as colorless crystals, ESIMS: m/z 123.1489 $[M+H]^+$. 1H NMR (600 MHz, $CDCl_3$) δ 9.80 (s, 1H, H-1), 9.65 (s, 1H, H-7), 7.77 (d, *J*=8.3 Hz, 2H, H-3,5), 6.94 (d, *J*=8.4 Hz, 2H, H-2,6).13C NMR (150 MHz, $CDCl_3$) δ 191.58(C-7), 163.37(C-1), 132.58(C-4), 128.96(C-2,6), 116.07(C-3,5).

**Cyclo-(L-Leu-D-Val) (4)**: $C_{11}H_{20}O_2N_2$ was obtained as white solid, $[\alpha]^{20}_D$ +5.2° (c 0.1, $CHCl_3$), ESIMS: m/z 235.1417 $[M+Na]^+$. $^1H$ NMR (600 MHz, $CDCl_3$) δ6.47 (s, 1H, H-1), 6.28 (s, 1H, H-4), 4.06 (m, 1H, H-3), 3.93 (m, 1H, H-6), 2.44 (m, 1H), 1.95-1.89 (m, 1H), 1.81 (m, 1H), 1.67-1.62 (m, 1H), 1.08 (d, *J*=7.1 Hz, 6H), 1.00 (d, *J* = 6.6 Hz, 6H). $^{13}C$ NMR (151 MHz,) δ168.85(C-5), 167.31(C-2), 60.35(C-3), 53.22(C-6), 43.83, 31.58, 24.40, 23.46, 21.13, 18.99, 16.54.

**Cyclo-(L-Pro-D-Leu) (5)**: $C_{11}H_{18}O_2N_2$ was obtained as white amorphous powder, $[\alpha]^{20}_D$ -130° (c 0.1, $CHCl_3$), ESIMS: m/z 233.1259 $[M+Na]^+$. $^1H$ NMR (400 MHz, $CDCl_3$) δ 4.12 (t, *J*=8.1 Hz, 1H, H-8a), 4.01 (dd, *J*=9.2, 3.2 Hz, 1H, H-3), 3.70-3.42 (m, 2H, H-6), 2.43-2.25 (m, 1H, H-8b), 2.21-1.97 (m, 3H, H-8a, 10a, 7b), 1.91 (d, *J*=19.4 Hz, 1H, H-7a), 1.79 (m, 1H), 1.61-1.49 (m, 1H), 0.98 (dd, *J*=17.5, 6.6 Hz, 6H).$^{13}C$ NMR (101MHz, $CDCl_3$) δ172.78(C-4), 168.90(C-1), 60.27(C-8a), 54.63(C-6), 46.43(C-6), 39.40, 29.06(C-8), 25.76, 23.65, 23.30(C-7), 22.21.

**Cyclo-(L-Pro-L-Leu) (6)**: $C_{11}H_{18}O_2N_2$ was obtained as white amorphous powder, $[\alpha]^{20}_D$ -85.4° (c 0.1, $CHCl_3$) ESIMS m/z 211.1433 $[M+H]^+$. $^1H$ NMR (600 MHz, $CDCl_3$) δ 6.26 (s, 1H, H-2), 4.10 (t, *J*=8.2 Hz, 1H, H-8a), 4.00 (dd, *J*=9.4, 3.6 Hz, 1H, H-3), 3.55 (m, 2H, H-6), 2.33 (m, 1H, H-8b), 2.12 (m, 1H, H-8a), 2.05 (m, 1H, H-10a), 2.02 (m, 1H, H-7b), 1.91 (m, 1H, H-7a), 1.73 (m, 1H), 1.51 (m, 1H), 0.99 (d, *J*=6.6 Hz, 3H), 0.94 (d, *J*=6.6 Hz, 3H).$^{13}C$ NMR (151 MHz, $CDCl_3$) δ 170.44(C-4), 164.36 (C-1), 59.10 (C-8a), 53.52 (C-3), 45.61(C-5), 38.69 , 28.21 (C-8), 24.76, 23.40, 22.86 (C-7), 21.35 .
