# Supplementary figures and images for: Active Secondary Metabolites from Root-Associated Endophytic Fungus Aspergillus tubingensis ZMGR14 and Their Activities Against Plant Pathogenic Fungi
Source: Biology (Basel). 2026 May 21;15(10):812. doi: 10.3390/biology15100812 (PMC13203383; doi:10.3390/biology15100812)

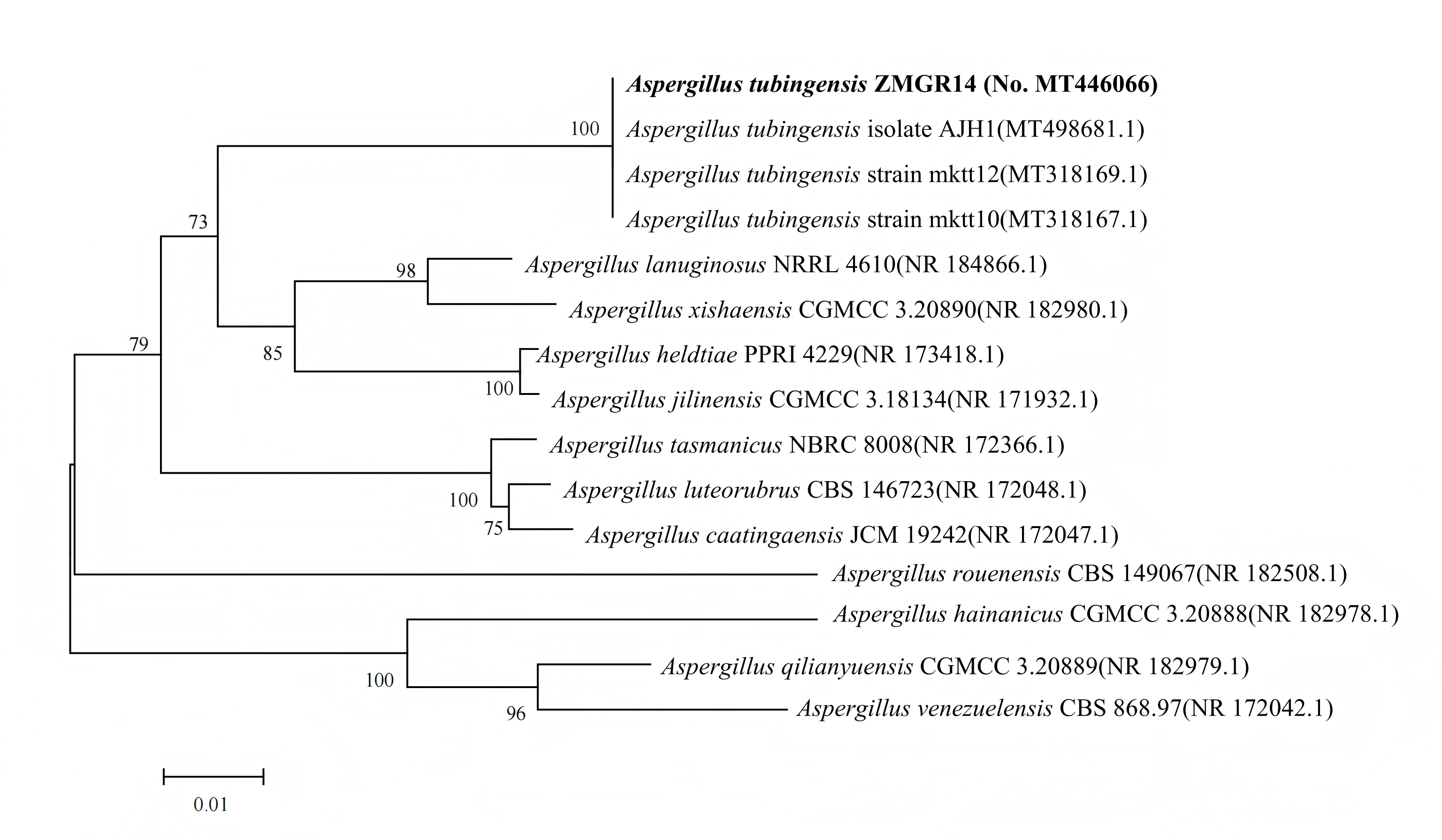

Supplement: Supplementary file 1 [file biology-15-00812-s001.zip › Figure S1.png]
